# Tuberculosis Recurrence and Mortality after Successful Treatment: Impact of Drug Resistance

**DOI:** 10.1371/journal.pmed.0030384

**Published:** 2006-10-03

**Authors:** Helen Cox, Yared Kebede, Sholpan Allamuratova, Gabit Ismailov, Zamira Davletmuratova, Graham Byrnes, Christine Stone, Stefan Niemann, Sabine Rüsch-Gerdes, Lucie Blok, Daribay Doshetov

**Affiliations:** 1 The University of Melbourne, Victoria, Australia; 2 Médecins Sans Frontières—Holland, Amsterdam, Netherlands; 3 Médecins Sans Frontières—Uzbekistan, Tashkent, Uzbekistan; 4 Ministry of Health, Nukus, Uzbekistan; 5 Centre for Molecular, Environmental, Genetic, and Analytic Epidemiology, The University of Melbourne, Victoria, Australia; 6 Department of Human Services, Victoria, Australia; 7 National Reference Centre for Mycobacteria, Borstel, Germany; 8 Royal Tropical Institute, Amsterdam, The Netherlands; University of California San Francisco, United States of America

## Abstract

**Background:**

The DOTS (directly observed treatment short-course) strategy for tuberculosis (TB) control is recommended by the World Health Organization globally. However, there are few studies of long-term TB treatment outcomes from DOTS programs in high-burden settings and particularly settings of high drug resistance. A DOTS program was implemented progressively in Karakalpakstan, Uzbekistan starting in 1998. The total case notification rate in 2003 was 462/100,000, and a drug resistance survey found multidrug-resistant (MDR) Mycobacterium tuberculosis strains among 13% of new and 40% of previously treated patients. A retrospective, observational study was conducted to assess the capacity of standardized short-course chemotherapy to effectively cure patients with TB in this setting.

**Methods and Findings:**

Using routine data sources, 213 patients who were sputum smear-positive for TB, included in the drug resistance survey and diagnosed consecutively in 2001–2002 from four districts, were followed up to a median of 22 months from diagnosis, to determine mortality and subsequent TB rediagnosis. Valid follow-up data were obtained for 197 (92%) of these patients. Mortality was high, with an average of 15% (95% confidence interval, 11% to 19%) dying per year after diagnosis (6% of 73 pansusceptible cases and 43% of 55 MDR TB cases also died per year). While 73 (74%) of the 99 new cases were “successfully” treated, 25 (34%) of these patients were subsequently rediagnosed with recurrent TB (13 were smear-positive on rediagnosis). Recurrence ranged from ten (23%) of 43 new, pansusceptible cases to six (60%) of ten previously treated MDR TB cases. MDR M. tuberculosis infection and previous TB treatment predicted unsuccessful DOTS treatment, while initial drug resistance contributed substantially to both mortality and disease recurrence after successful DOTS treatment.

**Conclusions:**

These results suggest that specific treatment of drug-resistant TB is needed in similar settings of high drug resistance. High disease recurrence after successful treatment, even for drug-susceptible cases, suggests that at least in this setting, end-of-treatment outcomes may not reflect the longer-term status of patients, with consequent negative impacts for patients and for TB control.

## Abstract 2

A retrospective, observational study was conducted to assess the effectiveness of a "DOTS" tuberculosis control program in Uzbekistan. High rates of disease recurrence were found among patients whose treatment had been initially successful.

## Editors' Summary

**Background.:**

Throughout history, tuberculosis (TB) has been a leading infectious cause of death—it kills about 2 million people every year. Until the 1940s, there was no effective treatment for TB, a chronic bacterial infection, usually of the lungs. Then, antibiotics active against the bacteria that cause TB—Mycobacterium tuberculosis—were introduced, and its incidence (the annual number of new cases) declined rapidly, particularly in developed countries. However, in the 1980s, there was a resurgence of TB, much of it driven by the HIV/AIDS epidemic—people with damaged immune systems are very susceptible to TB—and the emergence of drug-resistant M. tuberculosis. In 1995, the World Health Organization instigated what it called “DOTS,” an international strategy for global TB control. Central to DOTS is directly observed standardized short-course drug treatment. To prevent relapse and the emergence of drug-resistant bacteria, TB patients have to take antibiotics regularly for six months, even if they feel better sooner. The DOTS approach ensures that they do this by having trained observers watch them swallow their medications.

**Why Was This Study Done?:**

DOTS aims to detect 70% of new cases of sputum smear-positive TB (sputum is mucus coughed up from the lungs) and to treat 85% of these patients successfully. Both a cure—a negative smear at the end of treatment—and completion of treatment are recorded as “treatment successes.” There is no requirement in DOTS to check for TB recurrence, and few studies have investigated the long-term outcomes of treatment, particularly in areas with a high TB burden or where there is a problem with multidrug-resistant TB. Such data are needed to indicate whether DOTS can deliver global TB control. In this study, the researchers asked how often TB recurred in patients treated in a DOTS program in Karakalpakstan, Uzbekistan, an area with one of the highest incidences of multidrug-resistant TB.

**What Did the Researchers Do and Find?:**

The researchers identified about 200 sputum smear-positive TB patients who were treated consecutively in the Karakalpakstan DOTS program in 2001–2002. For most of the patients, follow-up data were available for an average of 22 months, a legacy of the pre-DOTS TB treatment system in Uzbekistan. The researchers found that, although three-quarters of new cases were “successfully” treated (i.e., close to the DOTS goal), a third of these “successes” were later re-diagnosed with TB. Recurrence of TB was particularly common among patients whose initial disease was multidrug resistant. Previous TB treatment was also associated with an increased risk of disease recurrence. Overall, nearly a quarter of the study patients died from TB during the follow-up period. Again, patients initially infected with multidrug-resistant TB fared particularly badly. Finally, only 65% of successfully treated patients were still alive and had not been re-diagnosed with TB 18 months after completion of their treatment.

**What Do These Findings Mean?:**

These high rates of disease recurrence and mortality suggest that DOTS might not be sufficient to control TB in areas like Karakalpakstan where the disease burden is high and multidrug-resistant infections are common. These poor long-term outcomes, note the researchers, are not hinted at by the end-of-treatment outcomes reported by the DOTS program. Limitations in the present study mean, however, that further studies are needed before these findings can be extrapolated to other settings. For example, the study used historical data so the researchers could not determine whether inadequate adherence to the DOTS program had contributed to the poor long-term outcome or whether disease recurrence was due to a relapse of the initial infection (which might indicate poor treatment adherence) or a new infection. Nevertheless, the current results warn against relying on end-of-treatment outcomes to judge the potential effectiveness of DOTS in controlling TB, and suggest that the expansion of DOTS-Plus, a supplement to DOTS for use where multidrug resistant TB is common, should be made a priority.

**Additional Information.:**

Please access these Web sites via the online version of this summary at http://dx.doi.org/10.1371/journal.pmed.0030384.

- US National Institute of Allergy and Infectious Diseases, patient fact sheet on tuberculosis
- US Centers for Disease Control and Prevention, information for patients and professionals on tuberculosis
- MedlinePlusencyclopedia entry on tuberculosis
- NHS Direct Online, patient information on tuberculosis from the UK National Health Service
- World Health Organization information on the global elimination of tuberculosis, including details of DOTS and DOTS-Plus
- Medécin sans Frontières; information on TB and other health issues in Karakalpakstan

## Introduction

Tuberculosis (TB) has arguably killed more human beings than any other disease throughout history. Into the 21st century it is still one of the leading infectious causes of death, killing at least 2 million people every year [1,2]. In 1993, the World Health Organization (WHO) declared TB a global emergency and subsequently launched the DOTS (directly observed treatment short-course) strategy to control TB. The aim of DOTS was to detect and treat cases of sputum smear-positive TB in order to reduce further transmission and control the spread of the disease.

DOTS is a management strategy consisting of five principles: government commitment, passive case detection based on sputum smear microscopy, DOTS chemotherapy treatment regimens, an uninterrupted drug supply, and a standardized recording and reporting system. DOTS is now the internationally accepted strategy for controlling TB, with 183 countries implementing it and 4.4 million DOTS cases reported in 2004 [2]. For control of TB, DOTS programs aim to detect 70% of incident cases and to successfully treat 85% of these. Treatment success is based on end-of-treatment outcomes [3], and as there is no standardized system of monitoring patients after treatment to confirm cure or record recurrent TB, published data are scant on rates of recurrence from routine DOTS programs, particularly in settings of high drug resistance.

Several studies suggest that patients infected with drug-resistant strains of Mycobacterium tuberculosis do poorly when placed on standard short-course chemotherapy. End-of-treatment outcomes in patients infected with multidrug-resistant (MDR) TB, defined as resistance to at least isoniazid and rifampicin, are reported to be no better than in the prechemotherapy era [4–7]. However, data are more limited on the impact of initial drug resistance on rates of relapse following cure. High recurrence, which can be either relapse of the same infection or reinfection, has been reported among small numbers of successfully treated patients diagnosed with MDR TB or with rifampicin-resistant TB [8–12]. These data suggest that in high drug-resistance settings, end-of-treatment outcomes may not represent cure and therefore may exaggerate the potential impact of DOTS on TB control. This retrospective, observational study aimed to assess recurrent TB and mortality following treatment in such a setting.

## Methods

### Setting

The Autonomous Republic of Karakalpakstan in Uzbekistan has a high TB burden (total case notifications 462/100,000/year) and high drug resistance (13% among new and 40% among previously treated patients) [13]. The DOTS strategy was implemented progressively in this region starting in 1998, in line with recommendations from WHO [3] and the International Union Against Tuberculosis and Lung Disease (IUATLD) [14], replacing the existing Soviet-style system of TB diagnosis and treatment. In 2003 it covered the total population of approximately 1.5 million. Support for DOTS implementation from Médecins Sans Frontières (MSF, Doctors without Borders) included infrastructural support for inpatient facilities and laboratories, extensive training and supervision, drug supply, and overall program monitoring. Treatment success for new smear-positive patients registered in 2002 was 74%.

### Study Design

A retrospective observational study of a cross-sectional sample of sputum smear-positive TB patients was used to determine the association between DOTS end-of-treatment outcomes, subsequent rediagnosis of active TB, death, and other predictors, including drug resistance.

### Definitions

DOTS treatment outcomes were defined according to WHO recommendations [3]; “cure” was defined as a negative sputum smear at the end of treatment and on one previous occasion among patients initially sputum smear-positive. Patients who completed treatment without confirmation of cure through sputum smear and without evidence of treatment failure were termed “treatment complete”. Treatment success includes these two categories. The term TB recurrence is used throughout this study to denote a recorded rediagnosis of TB (either sputum smear-positive or -negative) after “successful” DOTS TB treatment. Recurrence rather than relapse is used, because the lack of DNA fingerprinting data at rediagnosis made it impossible to determine whether recurrences were due to relapse of the same infection or reinfection with a new strain. Smear-negative recurrence was the term used when a patient with a negative smear microscopy result was started on the DOTS retreatment regimen. This decision was made routinely in the program only after review of the patient file by a treatment committee, which included MSF clinicians. Death was recorded for a patient who died for any reason either during treatment or subsequently.

Terms used to describe resistance in the causative M. tuberculosis strains are: monoresistant, resistant to one first-line drug; polyresistant, resistant to more than one first-line drug, but not MDR; MDR, resistant to at least rifampicin and isoniazid; and pansusceptible, susceptible to all five first-line drugs.

### Study Population

The study population included all patients, recruited as previously described [13] for a cross-sectional survey of drug resistance. Briefly, all patients from four districts in Karakalpakstan, diagnosed as sputum smear-positive within the DOTS program between July 2001 and January 2002, were sequentially requested to participate. Overall, 68% of eligible patients were recruited. The main reasons for nonrecruitment were refusal to participate (due to initial poor communication), patient default before sputum collection for drug susceptibility testing (DST), and logistic constraints in the timely collection and transport of sputum samples. The nonparticipants were not significantly different in terms of age, sex, or district, and the final sample was considered representative of TB patients diagnosed and treated under the DOTS strategy in Karakalpakstan.

Written informed consent was obtained from patients prior to inclusion in the initial drug resistance survey, and the observational study was approved by the University of Melbourne Human Research Ethics Committee, the MSF International Ethical Review Board, and the Ethical committee of the Ministry of Health in the Republic of Uzbekistan. Further individual patient consent was not obtained for the retrospective observational study.

### DOTS TB Treatment

Patients were treated, irrespective of drug resistance, using one of two standard regimens [3]. The WHO category 1 regimen, for new patients, consisted of 2 mo of daily isoniazid, rifampicin, pyrazinamide, and ethambutol (with or without streptomycin) followed by 4 mo of isoniazid and rifampicin, three times weekly. The category 2 regimen, for previously treated patients, consisted of all five drugs daily for 3 mo minus streptomycin for the last month, followed by 5 mo of isoniazid, rifampicin, and ethambutol three times weekly. All patients were hospitalized during the intensive phase of treatment and received doses in the continuation phase ostensibly under direct observation by health care workers.

### Data Sources

Patients were retrospectively tracked through two data sources. The first was the DOTS database, a computerized database containing registration, treatment progress, and outcome data for all patients registered in Karakalpakstan. The data contained in the database is entered centrally from the paper-based registration books maintained in each district. The registration and recording system is based on that recommended by the IUATLD [14].

Second, patients were tracked through a paper-based TB patient record system (ambulatory cards), independent from DOTS and continued from the pre-DOTS TB treatment system. This system was continued because DOTS was not, at the time, the national TB strategy for Uzbekistan, and therefore national-level reporting was still required under the old system of patient classification and outcome definition [15]. Patients were therefore seen regularly as outpatients after DOTS treatment, regardless of clinical status, for long periods of time, sometimes up to many years, with the ambulatory cards updated regularly.

Death data were obtained from both the ambulatory cards and separate “death registers” both held in each district. Death data were subsequently verified by the Statistics Division of the Ministry of Health for Karakalpakstan. Patients were classified as lost to observation if there was no subsequent DOTS registration and if no updated ambulatory card was available, or if health care workers reported that the patient had left Karakalpakstan.

### Laboratory Methods

Sputum microscopy analysis was conducted routinely through a network of DOTS TB laboratories utilizing the Ziehl-Nielsen technique, following recommendations from WHO/IUATLD [14,16]. Quality assurance was based on the blinded rechecking of randomly selected sputum smears and was within acceptable limits throughout the period included in this study.

DST and DNA fingerprinting (spoligotyping) was conducted on M. tuberculosis strains obtained from patients at initial diagnosis, but not at rediagnosis during the study period. Cultures were obtained from sputum samples sent directly from Karakalpakstan to the supranational reference laboratory in Borstel, Germany and subsequently tested against the five first-line drugs used in the DOTS program. Primary isolation and culture of mycobacterial isolates were performed as described elsewhere [17]. DST was performed on Löwenstein-Jensen media by the proportion method. If growth was insufficient, DST was conducted using the modified proportion method on a BACTEC 460TB (Becton Dickinson Microbiology Systems, Cockeysville, Maryland, United States). Extraction of genomic DNA was performed according to a standardized protocol [18]. All isolates were analyzed by the spoligotyping technique to identify Beijing genotype isolates [19].

### Statistical Analysis

Treatment outcomes for different categories of patients were compared using Pearson's Chi^2^ test, with *p* < 0.05 considered formally significant. Recurrent TB after successful treatment was assessed by survival curves, calculated using the life table method with the Wilcoxon (Gehan) method used to compare curves. Multivariate models of survival were based on Cox's proportional hazard model, with the Grambsch-Therneau test of the proportional hazards assumption. Factors entered into models included: treatment success, categories of drug resistance, previous TB treatment, sex, infection with M. tuberculosis Beijing strain, and rural versus city residence. Interaction terms were introduced when suggested by stratified analyses or where the investigators believed they were plausible. Final models were generated using a backward stepwise procedure: variables were removed depending on the likelihood ratio test and after considering changes to other estimates. Weibull regression was used to test for changing hazard over time. Multivariate analysis of factors predicting unsuccessful DOTS treatment used relied on logistic regression with a similar variable selection procedure and the same factors listed above. Analyses were performed with either Epi-Info software (version 6.04d, Centers for Disease Control, Atlanta, Georgia, United States) or Stata (release 7; Stata Statistical Software, College Station, Texas, United States).

## Results

### Study Sample

Valid observational data was obtained up to 30 September 2003 for 197 (92%) of the 213 patients included in the drug resistance survey [13]. This was a median of 22 mo after diagnosis (range 19–27). Patients were more often male (64%), the mean age was 35 y (range 13–70), 40% lived in rural districts, and 50% of patients reported previous TB treatment of more than 1 mo duration.

### DOTS Treatment Outcomes

DOTS end-of-treatment outcomes were similar to that seen in Karakalpakstan as a whole; 74% (73/99) of new and 46% (45/98) of previously treated cases were successfully treated (Chi^2^ = 15.9; *p* < 0.0001) (Table 1). Overall, 29% of successfully treated patients did not have sputum smear confirmation of cure at the end of treatment, and were therefore classified as treatment completed. Patients providing saliva samples instead of sputum were also classified as treatment completed.

**Table 1:**
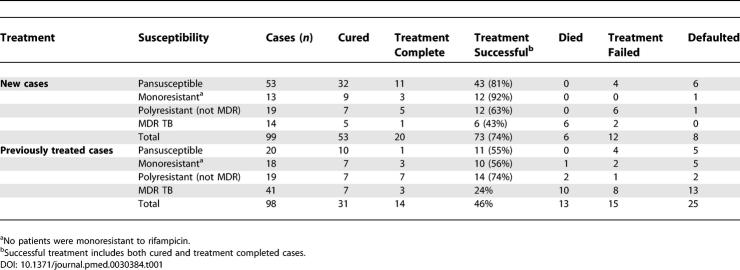
DOTS End-of-Treatment Outcomes by Categories of Drug Resistance in Terms of Previous TB Treatment

Among the 66 new patients infected with pansusceptible or monoresistant strains, 55 (83%) were classified as successfully treated. Patients infected with MDR M. tuberculosis did poorly, 43% (6/14) treatment success for new and only 24% (10/41) for those previously treated. Patients infected with M. tuberculosis strains resistant to more than one drug, but not both isoniazid and rifampicin (defined here as polyresistance) had intermediate treatment success (63% among new, and 74% among previously treated).

### Mortality

Mortality at the time of assessment was high, with 48 (24%) of the 197 patients dead at the time of follow-up. This equates to 15% of patients dying per year after diagnosis, ranging from 2% among new pansusceptible cases to 69% in the small number of new, MDR TB cases. Of the 33 patients who defaulted from treatment, 12 (36%) had died at the time of follow-up. Similarly, 11 (41%) of the 27 patients defined as treatment failures had died.

Overall, 96% of these deaths were recorded as being caused by TB, with many deaths occurring within TB hospitals, although cause of death was not verified by autopsy.

### Recurrent TB

Among the 118 patients who were successfully treated, 42 (36%) were rediagnosed with active TB during the observation period, with 52% of these rediagnoses sputum smear positive (Table 2). Patients classified as treatment completed had significantly higher smear-positive recurrence than those classified as cured (Chi^2^ = 5.9; *p* = 0.02), but no difference in overall recurrence (Chi^2^ = 2.7; *p* = 0.1). Among successfully treated cases, TB recurrence (both smear-positive and overall) increased significantly with increasing drug resistance (Chi^2^ = 6.0; *p* = 0.01). Interestingly, there were no significant differences in TB recurrence between new and previously treated cases who were successfully treated (Table 2).

**Table 2:**
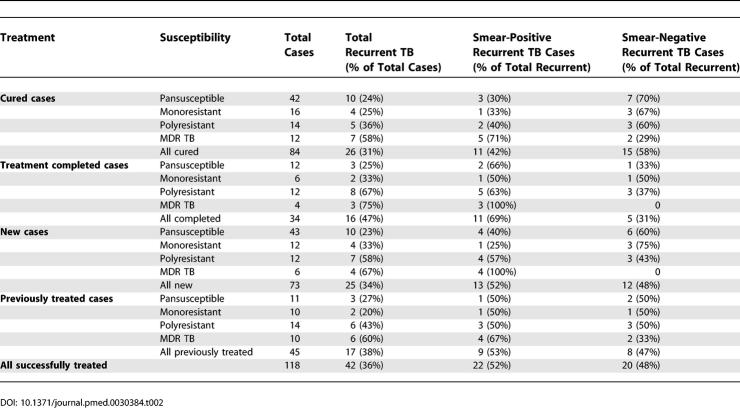
Recurrent TB by Drug Susceptibility among Successfully Treated Patients (*n* = 118), Stratified by Previous TB Treatment and by Cure and Treatment Completion

**Figure 1:**
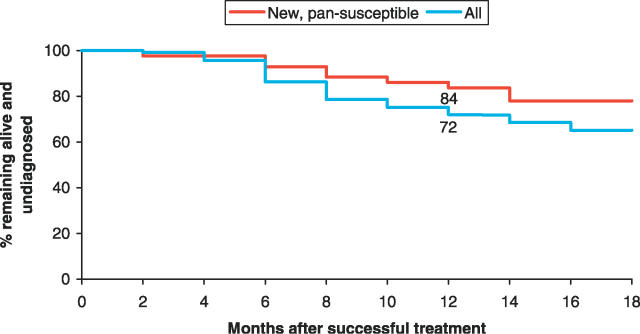
Continued Treatment Success over Time for New Pansusceptible Cases Compared to the Complete Study Sample Shown are cumulative percentages of successfully treated patients remaining alive and undiagnosed over time (life table method) for the complete study sample (*n* = 118) and for the subgroup of new, pansusceptible cases (*n* = 43). At 12 mo post-treatment 84% of new, pansusceptible cases were still alive and free of TB compared to 72% in the complete sample.

**Figure 2:**
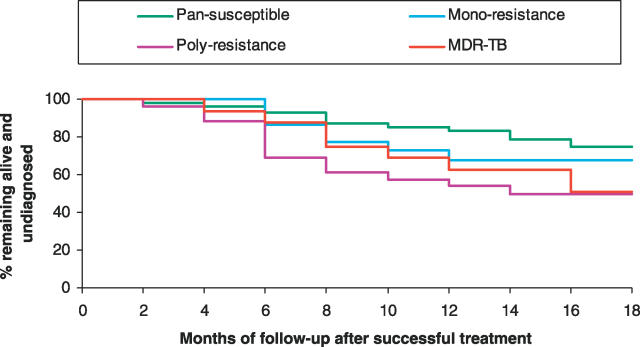
Effect of Drug Resistance on Continued Treatment Success over Time Shown are percentages of successfully treated patients remaining alive and undiagnosed over time (life table method) by categories of drug resistance

Many patients diagnosed with recurrent TB were subsequently reported to have died. Using the life table method, by 18 mo post-treatment only 65% of successfully treated patients were still alive and had not been rediagnosed with active TB (Figure 1). The rate of rediagnosis and/or death appeared to increase significantly across the observation period (*p* = 0.02), equating to a 1.3-fold increased hazard (95% confidence interval [CI], 1.04 to 1.76) at 12 mo compared to 6 mo. When stratified by drug resistance, disease-free survival at 18 mo ranged from 93% among those infected with pansusceptible M. tuberculosis strains to 56% among those with MDR TB (Figure 2).

Among the subgroup of 43 new patients infected with pansusceptible M. tuberculosis strains and successfully treated, recurrence of active TB remained significant. Four and six patients were rediagnosed with smear-positive and -negative disease, respectively, with two deaths. By 18 mo after treatment, only 78% remained alive and undiagnosed using the life table method of survival analysis (Figure 1).

### Factors Associated with Mortality and Recurrent TB

With time to death after diagnosis as an outcome, patients who were successfully treated displayed a significantly different survival curve compared to those with unsuccessful treatment outcomes (treatment failure, default, or death). Therefore, in order to determine factors contributing to mortality, the data were stratified by treatment success. After stratification, infection with MDR M. tuberculosis was the only significant predictor of mortality (hazard ratio, 3.0; 95% CI, 1.6 to 5.4).

As DOTS treatment outcome is clearly an important predictor of mortality, a logistic regression analysis was used to predict unsuccessful treatment. On univariate analysis, previous TB treatment, MDR TB, M. tuberculosis Beijing genotype, and city residence were significant predictors. However, in the multivariate model, Beijing genotype was no longer significant. The three factors predicting unsuccessful treatment were: MDR TB (odds ratio, 5.6; 95% CI, 2.6 to 11.8), previous treatment (2.2; 95% CI, 1.1 to 4.3), and city residence (3.1; 95% CI, 1.6 to 6.2).

A separate model examined time from successful treatment to death and/or rediagnosis among the 118 patients who were successfully treated. On univariate analysis, polyresistance and MDR TB were significant. In the multivariate model, both polyresistance and MDR TB remained significant. Female sex was a risk factor when combined with city residence (interaction *p* = 0.003), although neither of these factors was separately associated with the outcome. Hazard ratios were: polyresistance (3.1; 95% CI, 1.5 to 6.3), MDR TB (2.7; 95% CI, 1.3 to 5.9) and females in the city (2.9; 95% CI, 1.5 to 5.5). When only mortality after successful treatment was assessed, there was no significant effect for city women.

## Discussion

This study has demonstrated extremely high mortality and high rates of disease recurrence after “successful” treatment among TB patients treated with standardized short-course chemotherapy under a routine DOTS program. Despite the lack of data differentiating relapse from reinfection, high recurrence in such settings of drug resistance suggests that DOTS programs will have limited impact in terms of reducing TB incidence. Overall, 15% of patients died per year after diagnosis, and 36% of successfully treated patients were rediagnosed with active TB. These poor post-treatment outcomes are not hinted at by the end-of-treatment outcomes for these patients, as commonly reported for DOTS programs. The 74% treatment success rate for newly diagnosed patients is comparable to that achieved in many of the high TB-burden countries defined by WHO [2].

Infection with MDR TB had a direct impact on mortality and, along with previous TB treatment and residence in the city, contributed to poor end-of-treatment outcomes in the DOTS program. Both MDR TB and drug polyresistance contributed to high rates of recurrence and mortality after successful treatment. Among those infected with MDR *M. tuberculosis,* the rate of recurrent disease after successful treatment was 63%, with the majority positive by sputum smear. This rate is higher than the 28% recurrence reported among 18 patients in Russia, where the median duration of follow-up was only 6.5 mo [8]. Importantly, disease recurrence in Karakalpakstan was also substantial among those infected with polyresistant *M. tuberculosis;* 50% were rediagnosed after successful treatment.

Control of TB, and especially the disease caused by drug-resistant M. tuberculosis strains, seems unlikely in the face of these results. This conclusion is in contrast to a recent study from southern Mexico, in which a standard DOTS program was shown to reduce incidence of both drug-susceptible and drug-resistant TB [20]. However, this setting in southern Mexico did not have a high TB incidence prior to the implementation of DOTS (42/100,000/year) and reports a 3% MDR TB level among new cases; these indices are considerably lower than most of the high TB-burden and high drug-resistance settings, including Karakalpakstan [2,21]. Additionally, it is likely that the patterns of M. tuberculosis drug resistance seen in Mexico are less complicated than those in Uzbekistan.

Although drug resistance is an important contributor to poor long-term outcomes in this context, other factors may be present; among new patients infected with pansusceptible or monoresistant M. tuberculosis strains, treatment success was 83%, close to the WHO target of 85% [3]. But subsequently, 25% of these people were diagnosed with active TB again in the 18 mo after finishing treatment, with 36% of these recurrences sputum smear-positive. This result would suggest that the long term “cure” rate is substantially less than the 83% reported at the end of treatment.

A central question arising from these data is whether these recurrences of disease were caused by true relapses or by reinfection with new strains of M. tuberculosis. If relapse is common, it suggests that patients who are declared to be “successfully treated” in the DOTS program are not actually cured. Such treatment failure could result from inadequate treatment regimens, which in turn could arise through drug resistance, poor adherence to treatment, poor drug quality, or treatment regimens inadequate for this population, independent of drug resistance. Alternatively, reinfection could be an important contributor to an apparently high treatment failure rate. High rates of reinfection might be caused by a high prevalence of infectious TB in the community, low levels of immunity among individuals completing TB treatment, HIV infection, or specific characteristics of circulating M. tuberculosis strains. Without DNA fingerprinting of strains at rediagnosis, it is not possible to determine the relative contributions of relapse and reinfection in this study. The data suggest, however, that the rate of TB recurrence (and death) is, at least, not decreasing throughout the observation period; since relapse is thought to predominate in the first 6 mo after treatment completion [22], this suggests that both mechanisms may be involved.

In Karakalpakstan, most patients are hospitalized for the intensive phase of treatment, maximizing adherence to treatment. This practice was confirmed in a survey conducted in 2002 [23]. Randomly selected patients on intensive and continuation phases of treatment were interviewed to determine treatment observation and adherence. While both adherence and observation were considered adequate during the intensive phase (more than 80% of patients were observed for more than 80% of doses), observation declined in the continuation phase, with only 41% of patients observed taking more than 80% of doses. However, only 12% of patients reported missing more than 20% of doses in the continuation phase, and many of these patients went on to be recorded as default from treatment. Although no drug resistance data were available, among 11 patients from this survey known to have had more than 80% of doses observed, four patients were subsequently rediagnosed with sputum smear-positive TB. Therefore, it seems that poor adherence may not wholly explain the high disease recurrence rates seen here. Rates of default from treatment are higher in the main city in Karakalpakstan, however, resulting in the finding that city residence contributes significantly to unsuccessful treatment. This is most likely the result of overcrowding in TB facilities and poorer motivation among health care workers. Such findings demonstrate that despite significant inputs, there remain problems in program quality that manifest in poor observation of treatment.

Another possible explanation for high relapse may be poor drug quality. However, the antituberculosis drugs used in the Karakalpakstan DOTS program were supplied by MSF and sourced through the International Dispensary Association up to 2002. Subsequently, drugs were and are supplied to the Ministry of Health in Uzbekistan through the German Development Bank. Although this problem is possible, because of its sources drug quality seems unlikely to be a major explanatory factor.

If relapse is significant and cannot be completely attributed to drug resistance, poor adherence, or poor drug quality, we are led to question the adequacy of the drug regimes themselves under routine program conditions. To date there have been only two observational studies assessing post-treatment outcomes from functioning DOTS programs (using DOTS-recommended short-course chemotherapy regimens); both of these studies in India demonstrate an 11% rate of smear- or culture-positive disease recurrence [24,25]. It is therefore possible that the recommended treatment regimens are inadequate in some populations, given the realities of programmatic conditions.

Studies utilizing DNA fingerprinting of M. tuberculosis strains have shown that reinfection contributes more substantially to recurrent TB than previously thought [26], with between 12% and 77% of cases attributable to reinfection [27–31]. It is therefore plausible that in the high-prevalence region of Karakalpakstan, with many infected persons harboring drug-resistant strains, reinfection may contribute substantially to TB recurrence.

The high rates of mortality and recurrent TB seen here would, in many settings, suggest a high prevalence of HIV infection. HIV prevalence is reported to be increasing rapidly in the Central Asian republics [32]. However, in Uzbekistan, HIV seems, at present, to be confined to illicit users of injected drugs, particularly in the larger cities of Tashkent and Samarkand. In Karakalpakstan, officially, there were four cases of HIV infection up to 2004, and these were all reported in 2003. Despite the lack of widespread, adequate HIV testing, the data suggest that HIV has not yet affected the TB epidemic in Karakalpakstan.

### Study Limitations

As this was an observational study of a functioning DOTS program, there are a number of limitations to the data available for analysis. Principal among these limitations is the lack of adequate data on treatment adherence. Although observation of doses is routinely recorded, the survey mentioned above found that these data did not accurately reflect the true situation; therefore it was not used to assess treatment adherence in the current analysis. It is therefore not possible to determine the exact contribution to disease recurrence and mortality due to poor adherence in this study.

The relatively high proportion of successfully treated patients classified as “treatment completed” may also be considered a study limitation. The lack of a valid sputum smear result at the end of treatment potentially suggests poor adherence to treatment and a failure to complete the full treatment regimen. However, in the majority of such cases, the lack of a valid result was due to samples being recorded as “saliva” rather than sputum and therefore not analyzed. This suggests that sputum collection at the end of treatment is not adequately supervised or that patients could not actually produce sputum, not that patients failed to finish treatment. In contrast, the significantly higher proportion of smear-positive recurrences among the treatment-completed cases does suggest poorer adherence and emphasizes the need to obtain bacteriologically verified cure at the end of treatment.

The use of a recorded rediagnosis of TB was considered to be valid in this study due to the completeness of record-keeping and patient follow-up, carried forward from the previous treatment strategy. However, the lower rate of rediagnosis, but not mortality, among rural women, suggests that access to rediagnosis may be less than ideal, at least for some patients. This effect would result in an underestimated rate of recurrent TB. Conversely, since only sputum smear microscopy was used for rediagnosis, sputum smear-negative rediagnoses may be doubtful. However, a treatment committee assessed smear-negative rediagnoses, and only those started on the DOTS retreatment regime were included in this analysis. Additionally, the proportion of smear-positive rediagnoses is similar to that seen among new patients at diagnosis in the DOTS program in Karakalpakstan, suggesting that overdiagnosis is not significant among patients with recurrent disease.

### Conclusion

This study has shown very poor post-treatment patient outcomes for individuals diagnosed and treated for active TB in a relatively well-functioning DOTS program. These data suggest that, at least in this setting, and potentially in other settings with similar drug resistance profiles, DOTS programs are not sufficient to cure a sufficient number of individuals or for TB control. The expansion of DOTS-Plus programs for the specific treatment of drug-resistant TB is therefore urgently needed, particularly in resource-poor settings. Further studies differentiating relapse and reinfection are also required to formulate appropriate interventions. Additionally this study highlights the need for similar investigations into long-term outcomes from functioning DOTS programs in other settings.

## Abbreviations

CI: confidence interval
DOTS: directly observed treatment short-course
DST: drug susceptibility testing
MDR: multidrug-resistant
MSF: Médecins Sans Frontières
OR: odds ratio
TB: tuberculosis
WHO: World Health Organization

## References

[pmed-0030384-b001] Corbett EL, Watt CJ, Walker N, Maher D, Williams BG (2003). The growing burden of tuberculosis: Global trends and interactions with the HIV epidemic. Arch Intern Med.

[pmed-0030384-b002] World Health Organization (2006). Global tuberculosis control: Surveillance, planning and financing. WHO/HTM/TB/2006.362.

[pmed-0030384-b003] World Health Organization (2003). Treatment of tuberculosis: Guidelines for national programmes. WHO/CDS/TB/2003.313.

[pmed-0030384-b004] Espinal MA, Kim SJ, Suarez PG, Kam KM, Khomenko AG (2000). Standard short-course chemotherapy for drug-resistant tuberculosis: Treatment outcomes in 6 countries. JAMA.

[pmed-0030384-b005] Lan NTN, Iademarco MF, Binkin NJ, Tung LB, Quy HT (2001). A case series: Initial outcome of persons with multidrug-resistant tuberculosis after treatment with the WHO standard retreatment regimen in Ho Chi Minh City, Vietnam. Int J Tuberc Lung Dis.

[pmed-0030384-b006] Seung KJ, Gelmanova IE, Peremitin GG, Golubchikova VT, Pavlova VE (2004). The effect of initial drug resistance on treatment response and acquired drug resistance during standardized short-course chemotherapy for tuberculosis. Clin Infect Dis.

[pmed-0030384-b007] Jindani A, Nunn AJ, Enarson DA (2004). Two 8-month regimens of chemotherapy for treatment of newly diagnosed pulmonary tuberculosis: International multicentre randomised trial. Lancet.

[pmed-0030384-b008] Migliori GB, Espinal M, Danilova ID, Punga VV, Grzemska M (2002). Frequency of recurrence among MDR-TB cases “successfully” treated with standardised short-course chemotherapy. Int J Tuberc Lung Dis.

[pmed-0030384-b009] Singla R, Al-Sharif N, Al-Sayegh MO, Osman MM, Shaikh MA (2002). Influence of anti-tuberculosis drug resistance on the treatment outcome of pulmonary tuberculosis patients receiving DOTS in Riyadh, Saudi Arabia. Int J Tuberc Lung Dis.

[pmed-0030384-b010] Mitchison DA, Nunn AJ (1986). Influence of initial drug resistance on the response to short-course chemotherapy of pulmonary tuberculosis. Am Rev Respir Dis.

[pmed-0030384-b011] Garcia-Garcia ML, Ponce de Leon A, Jimenez-Corona ME, Jimenez-Corona A, Palacios-Martinez M (2000). Clinical consequences and transmissibility of drug-resistant tuberculosis in southern Mexico. Arch Intern Med.

[pmed-0030384-b012] Quy HTW, Lan NTN, Borgdorff MW, Grosset J, Linh PD (2003). Drug resistance among failure cases of tuberculosis: Is the standard re-treatment regimen adequate?. Int J Tuberc Lung Dis.

[pmed-0030384-b013] Cox HS, Orozco JD, Male R, Ruesch-Gerdes S, Falzon D (2004). Multidrug-resistant tuberculosis in central Asia. Emerg Infect Dis.

[pmed-0030384-b014] Enarson D, Rieder HL, Arnadottir T, Trébucq A (2000). Management of tuberculosis, a guide for low income countries.

[pmed-0030384-b015] Drobniewski F, Tayler E, Ignatenko N, Paul J, Connolly M (1996). Tuberculosis in Siberia: 2. Diagnosis, chemoprophylaxis and treatment. Tuber Lung Dis.

[pmed-0030384-b016] World Health Organization (2002). Training modules: Managing TB at a district level. WHO/CDS/TB/2002.310.

[pmed-0030384-b017] Kent P, Kubica G (1985). Public health mycobacteriology: A guide for the level III laboratory.

[pmed-0030384-b018] van Embden JD, Cave MD, Crawford JT, Dale JW, Eisenach KD (1993). Strain identification of Mycobacterium tuberculosis by DNA fingerprinting: Recommendations for a standardized methodology. J Clin Microbiol.

[pmed-0030384-b019] Kamerbeek J, Schouls L, Kolk A, van Agterveld M, van Soolingen D (1997). Simultaneous detection and strain differentiation of Mycobacterium tuberculosis for diagnosis and epidemiology. J Clin Microbiol.

[pmed-0030384-b020] DeRiemer K, Garcia-Garcia L, Bobadilla-del-Valle M, Palacios-Martinez M, Martinez-Gamboa A (2005). Does DOTS work in populations with drug-resistant tuberculosis?. Lancet.

[pmed-0030384-b021] World Health Organization (2004). Anti-tuberculosis drug resistance in the world: Third global report.

[pmed-0030384-b022] Sonnenberg P, Murray J, Glynn JR, Shearer S, Kambashi B (2001). HIV-1 and recurrence, relapse, and reinfection of tuberculosis after cure: A cohort study in South African mineworkers. Lancet.

[pmed-0030384-b023] Médecins Sans Frontières Uzbekistan (2003). Patient survey of treatment adherence and observation: Karakalpakstan 2002.

[pmed-0030384-b024] Vijay S, Balasangameswara VH, Jagannatha PS, Saroja VN, Kumar P (2004). Treatment outcome and two & half years follow-up status of new smear positive patients treated under RNCTP. Indian J Tuberc.

[pmed-0030384-b025] Thomas A, Gopi PG, Santha T, Chandrasekaran V, Subramani R (2005). Predictors of relapse among pulmonary tuberculosis patients treated in a DOTS programme in South India. Int J Tuberc Lung Dis.

[pmed-0030384-b026] Lambert ML, Hasker E, Van Deun A, Roberfroid D, Boelaert M (2003). Recurrence in tuberculosis: Relapse or reinfection?. Lancet Infect Dis.

[pmed-0030384-b027] Verver S, Warren RM, Beyers N, Richardson M, van der Spuy GD (2005). Rate of reinfection tuberculosis after successful treatment is higher than rate of new tuberculosis. Am J Respir Crit Care Med.

[pmed-0030384-b028] Das S, Chan SL, Allen BW, Mitchison DA, Lowrie DB (1993). Application of DNA fingerprinting with IS986 to sequential mycobacterial isolates obtained from pulmonary tuberculosis patients in Hong Kong before, during and after short-course chemotherapy. Tuber Lung Dis.

[pmed-0030384-b029] Das S, Paramasivan CN, Lowrie DB, Prabhakar R, Narayanan PR (1995). IS6110 restriction fragment length polymorphism typing of clinical isolates of Mycobacterium tuberculosis from patients with pulmonary tuberculosis in Madras, south India. Tuber Lung Dis.

[pmed-0030384-b030] Sahadevan R, Narayanan S, Paramasivan CN, Prabhakar R, Narayanan PR (1995). Restriction fragment length polymorphism typing of clinical isolates of Mycobacterium tuberculosis from patients with pulmonary tuberculosis in Madras, India, by use of direct-repeat probe. J Clin Microbiol.

[pmed-0030384-b031] van Rie A, Warren R, Richardson M, Victor TC, Gie RP (1999). Exogenous reinfection as a cause of recurrent tuberculosis after curative treatment. N Engl J Med.

[pmed-0030384-b032] Godinho J, Novotny T, Tadesse H, Vinokur A (2004). HIV/AIDS and tuberculosis in Central Asia. World Bank Working Paper No. 20.

